# Construction and Comparison of Different Models in Detecting Prostate Cancer and Clinically Significant Prostate Cancer

**DOI:** 10.3389/fonc.2022.911725

**Published:** 2022-07-12

**Authors:** Yongheng Zhou, Wenqiang Qi, Jianfeng Cui, Minglei Zhong, Guangda Lv, Sifeng Qu, Shouzhen Chen, Rongyang Li, Benkang Shi, Yaofeng Zhu

**Affiliations:** ^1^ Department of Urology, Qilu Hospital of Shandong University, Jinan, China; ^2^ Department of Thoracic Surgery, Qilu Hospital of Shandong University, Jinan, China

**Keywords:** prostate cancer, prostate health index, diagnosis, multiparametric magnetic resonance imaging, models

## Abstract

**Background:**

With the widespread adoption of prostatic-specific antigen (PSA) screening, the detection rates of prostate cancer (PCa) have increased. Due to the low specificity and high false-positive rate of serum PSA levels, it was difficult to diagnose PCa accurately. To improve the diagnosis of PCa and clinically significant prostate cancer (CSPCa), we established novel models on the basis of the prostate health index (PHI) and multiparametric magnetic resonance imaging (mpMRI) in the Asian population.

**Methods:**

We retrospectively collected the clinical indicators of patients with TPSA at 4–20 ng/ml. Furthermore, mpMRI was performed using a 3.0-T scanner and reported in the Prostate Imaging Reporting and Data System version 2.1 (PI-RADS). Univariable and multivariable logistic analyses were performed to construct the models. The performance of different models based on PSA derivatives, PHI derivatives, PI-RADS, and a combination of PHI derivatives and PI-RADS was evaluated.

**Results:**

Among the 128 patients, 47 (36.72%) patients were diagnosed with CSPCa and 81 (63.28%) patients were diagnosed with non-CSPCa. Of the 81 (63.28%) patients, 8 (6.25%) patients were diagnosed with Gleason Grade 1 PCa and 73 (57.03%) patients were diagnosed with non-PCa. In the analysis of the receiver operator characteristic (ROC) curves in TPSA 4–20 ng/ml, the multivariable model for PCa was significantly larger than that for the model based on the PI-RADS (*p* = 0.004) and that for the model based on the PHI derivatives (*p* = 0.031) in diagnostic accuracy. The multivariable model for CSPCa was significantly larger than that for the model based on the PI-RADS (*p* = 0.003) and was non-significantly larger than that for the model based on the PHI derivatives (*p* = 0.061) in diagnostic accuracy. For PCa in TPSA 4–20 ng/ml, a multivariable model achieved the optimal diagnostic value at four levels of predictive variables. For CSPCa in TPSA 4–20 ng/ml, the multivariable model achieved the optimal diagnostic value at a sensitivity close to 90% and 80%.

**Conclusions:**

The models combining PHI derivatives and PI-RADS performed better in detecting PCa and CSPCa than the models based on either PHI or PI-RADS.

## Introduction

Prostate cancer (PCa) is the second most common malignancy in men and the fifth leading cause of death in the world, with an estimated 1.41 million new cases in 2020 (1). Although the mortality and incidence of PCa are the highest in Northern Europe and the United States, the incidence of PCa has recently increased in Asia (2). With the widespread adoption of PSA screening, the detection rates of PCa have increased. However, due to the low specificity and high false-positive rate of serum PSA levels, it was difficult to diagnose PCa accurately (3, 4). Scholars established different models or nomograms for detecting PCa and/or clinically significant prostate cancer (CSPCa) (5–8). Due to the incidence rate of the regional characteristics of PCa, many models developed by the researchers in Europe and the United States are not applicable to the Asian population (9). It is necessary to develop diagnostic models or nomograms on the basis of the Asian population. Patients could profit from these diagnostic models and nomograms, which could avoid unnecessary prostate biopsy that may cause severe complications and even death (10).

The Prostate Health Index (PHI), which was determined by total prostate-specific antigen (TPSA), free prostate-specific antigen (fPSA), and isoform [-2]pro-prostate-specific antigen (P2PSA), improves the detection rate of PCa in some studies (11). Meanwhile, other studies that suggest multiparametric magnetic resonance imaging (mpMRI) also facilitate the detection of PCa (12).

In the current studies, limited serum biomarkers, such as the ratio of free to total PSA (f/T), prostatic-specific antigen density (PSAD), and urine spermine, have been used to help improve the diagnosis rate of PCa with PSA levels between 4 and 20 ng/ml (13–15). Although the most common indicator of f/T could improve the diagnostic efficiency with TPSA in 4–10 ng/ml, the specificity of this indicator was still not high. In different studies, the sensitivity and specificity were inconsistent in the same cutoff value of f/T (15, 16), which showed that diagnostic accuracy for PCa still needs to be improved with other indicators or models. Studies combining PHI with mpMRI at TPSA levels of 4–20 ng/ml have been limited for detecting CSPCa in Asian men. To confirm the clinical efficiency of PHI and mpMRI in the Asian population, we established a novel model on the basis of PHI, mpMRI, and other PSA derivatives.

## Methods and Patients

### Study Population

This retrospective study was approved by the Institutional Ethics Review Board of QILU Hospital of Shandong University (KYLL-202111-107), and a waiver of informed consent was obtained. The study was conducted in accordance with the Declaration of Helsinki. Between September 2020 and November 2021, 128 patients who underwent mpMRI examination and transperineal prostate biopsy were included. All patients were biopsy-naïve in this cohort. The inclusion criteria of the patients were as follows: (I) PSA 4–20 ng/ml; (II) serum samples were indwelled, and mpMRI was performed before biopsy; and (III) other clinical information was completed. The exclusion criteria of the patients were as follows: (I) abnormal white blood cells or platelets in routine blood examination; (II) urinary tract infection or prostatitis; (III) prostate surgery (such as transurethral resection of the prostate) was performed before biopsy; and (IV) incomplete clinical information.

### The Collection of Clinical Variables

Clinical data included age, body mass index (BMI), medical history of diabetes mellitus (DM) and hypertension, the number of cores applied to the target lesion, serum PSA derivatives (such as TPSA and fPSA), serum PHI and serum P2PSA levels, prostate volume (PV), PI-RADS v2.1 scores, and prostate biopsy Gleason score (PBGS). PV was measured by a urogenital radiologist with at least 2 years of experience from an MRI system and calculated using the following the prolate ellipsoid formulation: PV = ([maximum anteroposterior diameter] * [maximum transverse diameter] * [maximum longitudinal diameter] * 0.52) (17). The f/T value was calculated by dividing fPSA by TPSA, PSAD was calculated by dividing TPSA by PV, and PHID was calculated by dividing PHI by PV. The possibility of PCa observed on mpMRI was explained by the Prostate Imaging Reporting and Data System (PI-RADS) v2.1 scores (18).

Before biopsy, all patients underwent mpMRI examinations, which were performed by an experienced uroradiologist using a 3.0-T scanner. The mpMRI scan protocol included T2-weighted imaging (T2WI), diffusion-weighted imaging (DWI), dynamic contrast-enhanced imaging (DCE), and apparent diffusion coefficient (ADC) mapping. Two urogenital radiologists with at least 2 years of experience in prostate mpMRI interpreted the image of the prostate mpMRI and reached a consensus on PI-RADS score according to PI-RADS v2.1.

Ultrasound-guided transperineal prostate biopsy for all patients was performed with the help of mpMRI cognitive fusion. The patients routinely underwent 12-core systematic biopsy under localized anesthesia and additional X-cores were applied to the target lesions, which were identified from those most suspicious lesions with PI-RADS ≥ 3. The biopsy specimens were interpreted and graded by two experienced uropathologists in line with International Society of Urological Pathology Consensus Conference guidelines.

### Statistical Analysis

Continuous variables are expressed as the median (IQR), and categorical variables are reported as numbers (percentages). The differences in continuous data were assessed using the Student’s *t*-test for normal data and the Mann–Whitney *U* test for skewed data. Ranked data were analyzed by using the Mann–Whitney *U* test. The chi-square test or Fisher’s exact test was used to analyze categorical data. Univariable and multivariable logistic analyses were performed to select statistically significant predictors of CSPCa and non-CSPCa (including GS: 3+3 and no-PCa) on biopsy. For some inflated and imbalanced ORs, we conducted the logarithmic transformation in univariable and multivariable logistic analyses. The basic model based on PSA derivatives was established, and the other models were established with PHI or PI-RADS on the basis of the basic model. Finally, the final model was established by adding PHI and PI-RADS into the basic model. Receiver operating characteristic (ROC) curves and the area under the curve (AUC) were used to evaluate the predictive ability of different models. The diagnostic indicators of different models, such as specificity, positive predictive value (PPV), negative predictive value (NPV), overall diagnostic accuracy (ODA), positive likelihood ratio (+LR), and negative likelihood ratio (–LR), were compared at four levels of predictive variables (at a sensitivity close to 95%, 90%, 85%, and 80%). A subgroup analysis was also performed to analyze the clinical value of f/T in TPSA 4–10 ng/ml. Statistical analyses were performed using SPSS V.25.0 (IBM Corp, Armonk, NY, USA) and R statistical software (Version 4.1.0). *p* < 0.05 was considered statistically significant. The DeLong test was used to compare the differences in AUC.

## Results

A total of 128 patients were included in the study cohort. The patient characteristics of the study participants are summarized in **Table 1**. Among the 128 patients, 47 (36.72%) patients were diagnosed with CSPCa, and 81 (63.28%) patients were diagnosed with non-CSPCa. Of the 81 (63.28%) patients, 8 (6.25%) patients were diagnosed with Gleason Grade (GG)1 PCa and 73 (57.03%) patients were diagnosed with non-PCa. Age, BMI, PHI, TPSA, PSAD, and PHID were significantly higher in patients with CSPCa than in those without CSPCa. The f/T (0.11 vs. 0.14, *p* < 0.001) and PV (34.32 vs. 46.68 ml, *p* < 0.001) were smaller in CSPCa patients than in patients without CSPCa (**Table 1**). No differences were found in the number of targeted cores between CSPCa and non-CSPCa (*p* = 0.442).

**Table 1:** Patients’ characteristics.

Characteristics	Total (*N* = 128)	CSPCa (*N* = 47)	non-CSPCa (*N* = 81)	*p*
Age [years], median (IQR)	67.0 (60.3–73.0)	68.0 (64.0–75.0)	66.0 (59.0–71.5)	0.044
BMI [kg/m^2^], median (IQR)	24.2 (22.7–26.4)	24.7 (23.7–26.6)	23.9 (22.0–26.0)	0.025
DM, *n* (%)	20.0 (15.6)	10.0 (21.3)	10.0 (12.3)	0.180
Hypertension, *n* (%)	62.0 (48.4)	26.0 (55.3)	36.0 (44.4)	0.235
PHI, median (IQR)	54.4 (42.6–79.0)	86.7 (63.0–114.1)	45.6 (38.6–70.9)	<0.001
P2PSA [pg/ml], median (IQR)	21.5 (14.2–30.8)	31.0 (19.6–45.3)	18.4 (12.3–24.8)	<0.001
TPSA [ng/ml], median (IQR)	8.7 (6.1–12.3)	11.7 (8.1–14.6)	8.0 (5.9–10.3)	<0.001
fPSA [ng/ml], median (IQR)	1.1 (0.9–1.5)	1.1 (0.8–1.5)	1.1 (0.9–1.5)	0.943
f/T, median (IQR)	0.13 (0.10–0.18)	0.11 (0.08–0.14)	0.14 (0.11–0.19)	<0.001
PV [ml], median (IQR)	41.4 (28.1–62.0)	34.3 (24.4–54.1)	46.7 (33.8–66.9)	0.005
Targeted cores, median (IQR)	4.0 (0–6.0)	4.0 (0–6.0)	4.0 (0–5.0)	0.442
PSAD [ng/ml^2^], median (IQR)	0.2 (0.1–0.3)	0.3 (0.2–0.5)	0.2 (0.1–0.3)	<0.001
PHID, median (IQR)	1.3 (0.8–2.5)	2.6 (1.3–3.7)	1.0 (0.6–1.6)	<0.001
PI-RADS, *n* (%)				<0.001
≤2	46 (35.9)	5 (10.6)	41 (50.6)	
=3	42 (32.8)	21 (44.7)	21 (25.9)	
≥4	40 (31.2)	21 (44.7)	19 (23.5)	

BMI, body mass index; DM, diabetes mellitus; PHI, prostate health index; P2PSA, isoform [-2]pro–prostate-specific antigen; TPSA, total prostate-specific antigen; fPSA, free prostate-specific antigen; f/T, free/Total prostate-specific antigen; PV, prostate volume; PSAD: prostate-specific antigen density; PHID, prostate health index density; PI-RADS, Prostate Imaging Reporting and Data System version 2.1; IQR, interquartile range.

CSPCa is defined as Gleason Grade ≥ 2 prostate cancer.

### Univariable Logistic Regression Analysis Showed the Predictors of PCa and CSPCa

Univariable logistic regression analysis showed that age, BMI, PHI, P2PSA, TPSA, f/T, PV, Log (PSAD), PHID, and PI-RADS were significant predictors for PCa and CSPCa (**Table 2**). The OR value of age and BMI showed that the risk of PCa and CSPCa increased when the patients were older and had a higher BMI (**Table 2**). PHI performed better than PHID and PSAD in predicting PCa and CSPCa. The risks of PCa and CSPCa were negatively correlated with PV and f/T, which both showed low diagnostic accuracy in predicting PCa and CSPCa (**Table 2**). For PI-RADS, the ORs of PCa and CSPCa were the largest, and they were extremely reliable risk factors for PCa and CSPCa (OR = 2.877 for PCa and OR = 2.493 for CSPCa) (**Table 2**).

**Table 2:** Univariable logistic regression analysis for the prediction of total and clinically significant prostate cancer (CSPCa).

Clinical Parameters	Univariable logistic regression						Multivariable regression analysis					
	Total PCa			CSPCa			Total PCa			CSPCa		
	OR (95%CI)	AUC (95% CI)	*p*	OR (95% CI)	AUC (95% CI)	*p*	Coefficient	OR(95% CI)	*p*	Coefficient	OR(95% CI)	*p*
**Intercept**	–	–	–	–	–	–	-13.903	NA	<0.001	-10.916	NA	<0.001
**Age**	1.062 (1.016–1.109)	0.634 (0.538–0.731)	0.008	1.049 (1.004–1.096)	0.607 (0.506–0.708)	0.032	–	–	–	–	–	–
**BMI**	1.164 (1.026–1.322)	0.635 (0.540–0.731)	0.019	1.143 (1.007–1.297)	0.619 (0.522–0.716)	0.038	0.250	1.285 (1.059–1.558)	0.011	0.185	1.204 (1.008–1.437)	0.040
**PHI**	1.059 (1.036–1.083)	0.842 (0.771–0.912)	<0.001	1.052 (1.032–1.073)	0.867 (0.801–0.932)	<0.001	0.061	1.063 (1.037–1.090)	<0.001	0.050	1.051 (1.029–1.073)	<0.001
**P2PSA**	1.079 (1.041–1.119)	0.730 (0.641–0.819)	<0.001	1.077 (1.040–1.115)	0.752 (0.662–0.843)	<0.001	–	–	–	–	–	–
**TPSA**	1.147 (1.045–1.260)	0.642 (0.543–0.740)	0.004	1.228 (1.109–1.360)	0.715 (0.621–0.809)	<0.001	–	–	–	–	–	–
**f/T**	0.000 (0.000–0.136)	0.349 (0.254–0.445)	0.010	0.000 (0.000–0.035)	0.312 (0.217–0.406)	0.004	–	–	–	–	–	–
**PV**	0.977 (0.962–0.993)	0.319 (0.225–0.413)	0.004	0.982 (0.967–0.998)	0.351 (0.249–0.452)	0.023	–	–	–	–	–	–
**Target cores**	1.027 (0.912–1.157)	0.546 (0.446–0.646)	0.661	1.016 (0.899–1.148)	0.540 (0.437–0.643)	0.804	–	–	–	–	–	–
**Log (PSAD)**	24.419 (5.418–110.062)	0.738 (0.651–0.825)	<0.001	38.039 (7.515–192.545)	0.763 (0.676–0.850)	<0.001	–	–	–	–	–	–
**PHID**	2.814 (1.834–4.317)	0.825 (0.753–0.897)	<0.001	2.371 (1.632–3.447)	0.816 (0.738–0.893)	<0.001	–	–	–	–	–	–
**PI-RADS**	2.877 (1.881–4.403)	0.766 (0.686–0.846)	<0.001	2.493 (1.658–3.748)	0.735 (0.650–0.820)	<0.001	1.231	3.424 (1.860–6.303)	<0.001	0.848	2.336 (1.388–3.930)	0.001

OR, odds ratio; 95% CI, 95% confidence intervals; AUC, area under the curve; CSPCa, Gleason Grade ≥ 2 prostate cancer; BMI, body mass index; DM, diabetes mellitus; PHI, prostate health index; P2PSA, isoform [-2]pro–prostate-specific antigen; TPSA, total prostate-specific antigen; fPSA, free prostate-specific antigen; f/T, free/Total prostate-specific antigen; PV, prostate volume; PSAD, prostate-specific antigen density; Log (PSAD), The logarithmic transformation of PSAD could balance the OR; PHID, prostate health index density; PI-RADS, Prostate Imaging Reporting and Data System version 2.1.NA, not applicable.

### Establishment of Different Multivariable Models Based on PSA Derivatives

In a stepwise logistic regression analysis, age, BMI, PHI, P2PSA, TPSA, f/T, PV, and PI-RADS were included to establish the models for the detection of PCa and CSPCa. Finally, BMI, PHI, and PI-RADS were included in the multivariable models for detection of PCa and CSPCa (**Table 2**). To evaluate the predictive ability of PHI, PI-RADS, and the combination of both for PCa and CSPCa, four multivariable models were developed based on the multivariable logistic regression analysis (**Table S1**). The Model A formulas (base model) for PCa and CSPCa were constructed based on the age, BMI, TPSA, log (f/T), and PV. The logarithmic transformation of the f/T was used to balance the ORs of the model. The Model B formulas for PCa and CSPCa were based on the PHI derivatives and the base model. The Model C formulas for PCa and CSPCa were based on PI-RADS and the base model. The Model D formulas for PCa and CSPCa were based on the combination of PHI derivatives and PI-RADS.

In ROC analysis in TPSA 4–20 ng/ml, Model D for PCa (AUC = 0.928, 95% CI: 0.884–0.972) was significantly larger than Model C (*p* = 0.004) and Model B (*p* = 0.031) in terms of diagnostic accuracy. The outcomes of Model D for CSPCa (AUC = 0.913, 95% CI: 0.862–0.963) was significantly larger than those for Model C (*p* = 0.003) in diagnostic accuracy (**Table S1**). In detecting total PCa in TPSA 4–20 ng/ml, the multivariable model could avoid 73.97% unnecessary biopsies with 95% sensitivity.

For the analysis of value of f/T in TPSA 4–10 ng/ml (19, 20), we performed a subgroup analysis, which reconstructed the four models (**Table 3**). No difference was found in the base models for PCa and CSPCa between the two groups (AUC = 0.809 in TPSA 4–20 ng/ml vs AUC = 0.819 in TPSA 4–10 ng/ml, *p* = 0.881; AUC = 0.812 in TPSA 4–20 ng/ml vs AUC = 0.772 in TPSA 4–10 ng/ml, *p* = 0.591). The AUC of Model C was higher in TPSA 4–10 ng/ml than in Model C in TPSA 4–20 ng/ml for PCa and CSPCa. The AUC of Model D was significantly higher than that of Model B in TPSA 4–10 ng/ml for PCa (AUC = 0.927 vs. 0.865, *p* = 0.025). The discrepancies and diagnostic efficacy of the different four models in TPSA 4–10 ng/ml are also shown in **Table 3**.

**Table 3A:** Multivariable analysis of the predictive value of the different models in the diagnosis of PCa in the PSA 4–10 ng/ml.

Total PCa	Model A		Model B		Model C		Model D	
	OR (95% CI)	*p*	OR (95% CI)	*p*	OR (95% CI)	*p*	OR (95% CI)	*p*
Age	1.097 (1.004–1.198)	0.041	1.111 (1.003–1.231)	0.044	1.018 (0.919–1.128)	0.728	1.029 (0.921–1.151)	0.611
BMI	1.446 (1.152–1.815)	0.001	1.395 (1.094–1.778)	0.007	1.629 (1.199–2.213)	0.002	1.619 (1.156–2.267)	0.005
TPSA	1.064 (0.743–1.522)	0.736	0.907 (0.599–1.372)	0.644	1.052 (0.683–1.621)	0.816	0.886 (0.535–1.467)	0.886
Log(f/T)	0.230 (0.008–6.880)	0.397	0.328 (0.007–15.548)	0.571	0.412 (0.009–18.018)	0.645	1.246 (0.011–140.641)	0.927
PV	0.969 (0.938–1.001)	0.061	0.980 (0.947–1.014)	0.238	0.965 (0.932–1.000)	0.049	0.978 (0.944–1.013)	0.218
PHI	NA	NA	1.043 (1.010–1.076)	0.010	NA	NA	1.049 (1.004–1.097)	0.031
PI-RADS	NA	NA	NA	NA	4.041 (1.850–8.830)	<0.001	4.281 (1.718–10.668)	0.002
AUC (95%CI)	0.819 (0.715–0.922)		0.865 (0.781–0.949)		0.901 (0.827–0.976)		0.927 (0.868–0.986)
*p* (Model A as referent)	/		0.159		0.014		0.006
*p* (Model B as referent)	/		/		0.282		0.025
*p* (Model C as referent)	/		/		/		0.163

Model A, multivariable model based on the TPSA derivatives (base model); Model B, multivariable model based on the combination of PHI derivatives and base model; Model C, multivariable model based on combination of the PI-RADS and base model; Model D, multivariable model based on the combination of PHI derivatives and PI-RADS; odds ratio; 95% CI, 95% confidence intervals; BMI, body mass index; TPSA, total prostate-specific antigen; f/T, free/Total prostate-specific antigen; PV, prostate volume; PHI, prostate health index; PI-RADS, Prostate Imaging Reporting and Data System version 2.1; PCa, prostate cancer.NA, not applicable.

**Table 3B:** Multivariable analysis of the predictive value of the different models in the diagnosis of CSPCa in the PSA 4–10 ng/ml.

Total CSPCa	Model A		Model B		Model C		Model D	
	OR (95% CI)	*p*	OR (95% CI)	*p*	OR (95% CI)	*p*	OR (95% CI)	*p*
Age	1.053 (0.965–1.149)	0.250	1.049 (0.954–1.153)	0.323	0.990 (0.894–1.095)	0.841	0.990 (0.889–1.102)	0.851
BMI	1.338 (1.067–1.679)	0.012	1.288 (1.021–1.624)	0.033	1.401 (1.069–1.837)	0.015	1.336 (1.016–1.758)	0.038
TPSA	1.357 (0.926–1.987)	0.117	1.316 (0.872–1.988)	0.191	1.356 (0.889–2.070)	0.158	1.347 (0.862–2.105)	0.191
Log(f/T)	0.194 (0.005–7.301)	0.375	0.230 (0.004–12.568)	0.230	0.413 (0.007–26.124)	0.676	0.484 (0.006–38.497)	0.745
PV	0.980 (0.948–1.013)	0.229	0.987 (0.953–1.022)	0.453	0.977 (0.943–1.012)	0.194	0.983 (0.948–1.019)	0.350
PHI	NA	NA	1.024 (1.001–1.046)	0.037	NA	NA	1.018 (0.994–1.042)	0.137
PI-RADS	NA	NA	NA	NA	2.872 (1.435–5.748)	0.003	2.593 (1.281–5.251)	0.008
AUC (95%CI)	0.772 (0.650–0.894)		0.830 (0.721–0.938)		0.851 (0.749–0.953)		0.870 (0.774–0.967)
*p* (Model A as referent)	/		0.083		0.084		0.031
*p* (Model B as referent)	/		/		0.650		0.285
*p* (Model C as referent)	/		/		/		0.313

Model A, multivariable model based on the TPSA derivatives (base model); Model B, multivariable model based on the combination of PHI derivatives and base model; Model C, multivariable model based on combination of the PI-RADS and base model; Model D, multivariable model based on the combination of PHI derivatives and PI-RADS; odds ratio; 95% CI, 95% confidence intervals; BMI, body mass index; TPSA, total prostate-specific antigen; f/T, free/Total prostate-specific antigen; PV, prostate volume; PHI, prostate health index; PI-RADS, Prostate Imaging Reporting and Data System version 2.1; CSPCa, clinically significant prostate cancer; defined as Gleason Grade ≥ 2 prostate cancer.NA, not applicable.

**Figure 1:**
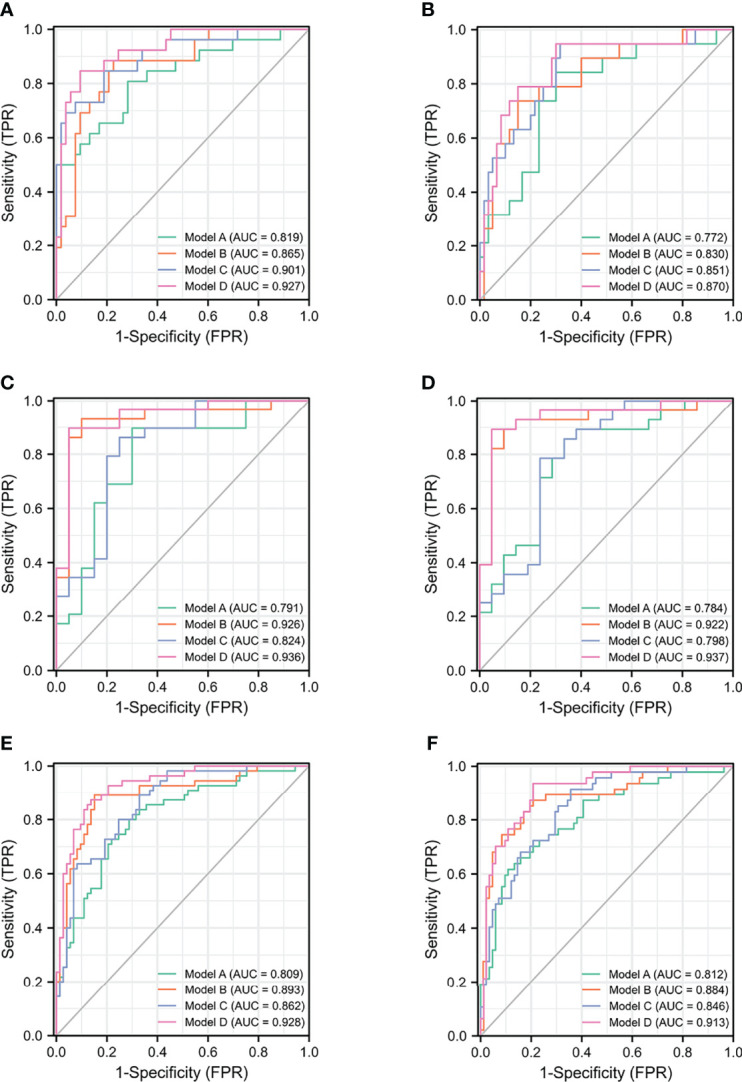
The ROC curves of models for PCa and CSPCa. **(A)** The models for PCa in TPSA 4–10 ng/ml. **(B)** The models for CSPCa in TPSA 4–10 ng/ml. **(C)** The models for PCa in TPSA 10–20 ng/ml. **(D)** The models for CSPCa in TPSA 10–20 ng/ml. **(E)** The models for PCa in TPSA 4–20 ng/ml. **(F)** The models for CSPCa in TPSA 4–20 ng/ml. Model A: multivariable model based on the TPSA derivatives (base model); Model B: multivariable model based on the combination of PHI derivatives and base model; Model C: multivariable model based on the combination of the PI-RADS and base model; Model D: multivariable model based on the combination of PHI derivatives and PI-RADS.

Based on the subgroup analysis in TPSA 10–20 ng/ml (**Table 4**), Model B had higher AUC values than Model C for PCa and CSPCa (AUC = 0.926 vs. 0.824, *p* = 0.064; AUC = 0.922 vs. 0.714, *p* = 0.035). The AUC of Model D was significantly higher than that of Model C for PCa and CSPCa (AUC = 0.936 vs. 0.824, *p* = 0.029; AUC = 0.937 vs. 0.714, *p* = 0.016), which is shown in **Table 4**. A comparison of the ROC curves for different models in different ranges of TPSA was presented in **Figure 1**, while curves of the correction for multiple comparisons of models are provided in **Figure S1**.

**Table 4A:** Multivariable analysis of the predictive value of the different models in the diagnosis of PCa in the PSA 10–20 ng/ml.

Total PCa	Model A		Model B		Model C		Model D	
	OR (95% CI)	*p*	OR (95% CI)	*p*	OR (95% CI)	*p*	OR (95% CI)	*p*
Age	1.104 (1.019–1.196)	0.015	1.064 (0.953–1.187)	0.270	1.084 (0.995–1.182)	0.066	1.024 (0.905–1.160)	0.704
BMI	0.975 (0.752–1.263)	0.848	1.153 (0.799–1.666)	0.447	0.938 (0.695–1.264)	0.672	1.102 (0.742–1.638)	0.630
TPSA	1.224 (0.921–1.625)	0.163	1.049 (0.756–1.457)	0.774	1.168 (0.864–1.578)	0.313	1.005 (0.714–1.415)	0.977
Log(f/T)	0.243 (0.001–48.355)	0.600	1.917 (0.001–4,116.621)	0.868	0.092 (0.000–19.520)	0.382	4.191 (0.002–9,069.647)	0.715
PV	0.975 (0.947–1.003)	0.080	0.983 (0.950–1.017)	0.316	0.982 (0.952–1.012)	0.241	0.988 (0.952–1.026)	0.534
PHI	NA	NA	1.075 (1.019–1.133)	0.008	NA	NA	1.081 (1.016–1.150)	0.014
PI-RADS	NA	NA	NA	NA	2.242 (0.977–5.146)	0.057	2.322 (0.729–7.399)	0.154
AUC (95%CI)	0.791 (0.656–0.926)		0.926 (0.841–1.000)		0.824 (0.699–0.949)		0.936 (0.862–1.000)
*p* (Model A as referent)	/		0.016		0.428		0.010
*p* (Model B as referent)	/		/		0.064		0.400
*p* (Model C as referent)	/		/		/		0.029

Model A, multivariable model based on the TPSA derivatives (base model); Model B, multivariable model based on the combination of PHI derivatives and base model; Model C, multivariable model based on combination of the PI-RADS and base model; Model D, multivariable model based on the combination of PHI derivatives and PI-RADS; OR, odds ratio; 95% CI, 95% confidence intervals; BMI, body mass index; TPSA, total prostate-specific antigen; f/T, free/Total prostate-specific antigen; PV, prostate volume; PHI, prostate health index; PI-RADS, Prostate Imaging Reporting and Data System version 2.1; PCa, prostate cancer; AUC, area under the curve.NA, not applicable.

**Table 4B:** Multivariable analysis of the predictive value of the different models in the diagnosis of CSPCa in the PSA 10–20 ng/ml.

Total CSPCa	Model A		Model B		Model C		Model D	
	OR (95% CI)	*p*	OR (95% CI)	*p*	OR (95% CI)	*p*	OR (95% CI)	*p*
Age	1.091 (1.011–1.176)	0.024	1.044 (0.938–1.162)	0.436	1.072 (0.989–1.162)	0.092	1.012 (0.898–1.140)	0.851
BMI	1.044 (0.813–1.339)	0.738	1.294 (0.890–1.881)	0.178	1.020 (0.777–1.339)	0.887	1.272 (0.853–1.896)	0.237
TPSA	1.286 (0.969–1.706)	0.082	1.107 (0.802–1.528)	0.537	1.243 (0.924–1.673)	0.150	1.070 (0.770–1.487)	0.686
Log(f/T)	0.248 (0.001–43.635)	0.597	3.945 (0.002–9,226.468)	0.729	0.117 (0.001–20.915)	0.417	7.349 (0.004–15,076.966)	0.608
PV	0.979 (0.953–1.006)	0.126	0.987 (0.956–1.020)	0.449	0.986 (0.958–1.014)	0.331	0.993 (0.958–1.029)	0.993
PHI	NA	NA	1.084 (1.024–1.148)	0.005	NA	NA	1.091 (1.022–1.164)	0.009
PI-RADS	NA	NA	NA	NA	1.925 (0.903–4.101)	0.090	1.941 (0.623–6.046)	0.253
AUC (95%CI)	0.784 (0.652–0.916)		0.922 (0.838–1.000)		0.798 (0.665–0.930)		0.937 (0.863–1.000)
*p* (Model A as referent)	/		0.014		0.714		0.007
*p* (Model B as referent)	/		/		0.035		0.137
*p* (Model C as referent)	/		/		/		0.016

Model A, multivariable model based on the TPSA derivatives (base model); Model B, multivariable model based on the combination of PHI derivatives and base model; Model C, multivariable model based on combination of the PI-RADS and base model; Model D, multivariable model based on the combination of PHI derivatives and PI-RADS; OR, odds ratio; 95% CI, 95% confidence intervals; BMI, body mass index; TPSA, total prostate-specific antigen; f/T, free/Total prostate-specific antigen; PV, prostate volume; PHI, prostate health index; PI-RADS, Prostate Imaging Reporting and Data System version 2.1; CSPCa, clinically significant prostate cancer; defined as Gleason Grade ≥ 2 prostate cancer; AUC, area under the curve.NA, not applicable.

The multivariable model for PCa and CSPCa and other predictors was analyzed with sensitivity, specificity, PPV, NPV, ODA, +LR, and –LR to compare the clinical utility and diagnostic value. The diagnostic indicators of different models were compared at four levels of predictive variables (at a sensitivity close to 95%, 90%, 85%, and 80%), which are shown in **Table S2**. For PCa in TPSA 4–20 ng/ml, Model D achieved the optimal specificity, PPV, NPV, +LR, −LR, and ODA at four levels of predictive variables. For CSPCa in TPSA 4–20 ng/ml, Model D achieved the optimal diagnostic value at a sensitivity close to 90% and 80%. The diagnostic value for PCa and CSPCa in TPSA 4–10 ng/ml and 4–20 ng/ml is also shown in **Table S2**.

## Discussion

This is the first retrospective study to evaluate PHI, PI-RADS, and a combination of both to predict PCa and CSPCa with TPSA in the range of 4–20 ng/ml before prostate biopsy in an Asian population. Age, BMI, TPSA, f/T, PV, PSAD, PHID, and PI-RADS were significant independent predictors of PCa and CSPCa in our study. Furthermore, four different models were established based on the TPSA derivatives, PHI derivatives, PI-RADS, and a combination of PHI and PI-RADS individually. In comparison with other models, the multivariable model based on the combination of PHI and PI-RADS showed the best diagnostic performance. The diagnostic accuracy of the model based on the combination of PHI and PI-RADS for CSPCa outperformed the model based on the TPSA derivatives and the model based on the PI-RADS, but showed non-significance with the model based on the PHI derivatives in TPSA 4–20 ng/ml (*p* = 0.061). However, the AUC of that multivariable model for CSPCa was higher than that of the other models. The AUC of the multivariable models for PCa and CSPCa was also both higher than those of PHI or PI-RADS alone and could avoid more unnecessary biopsies. Subgroup analyses were performed in TSA levels of 4–10 ng/ml and 10–20 ng/ml. In detecting PCa and CSPCa, Model D displayed the highest AUC of both subgroups. In order to determine whether f/T can improve the diagnostic value of the models in TPSA 4–10 ng/ml, we rebuilt these four models and compared their performance. Despite the lack of statistical significance, we observed that there was an increase for detecting PCa in the AUC of TPSA 4–10 ng/ml when comparing the two base models. The significant discrepancy between Model D and Model B showed that the PI-RADS indicator was more meaningful in the population with TPSA in the range of 4–10 ng/ml. Among the population with TPSA levels between 10 and 20 ng/ml, the AUC of Model D was significantly higher than that of Model C for PCa and CSPCa, indicating that PHI may improve the detection rates in these populations.

Some studies have shown that the application of PHI or mpMRI alone for the detection of aggressive PCa could reduce unnecessary biopsies before biopsy (12, 21). De la Calle et al. proposed that the diagnostic accuracy of PHI was superior to PSA and fPSA and could avoid 41% of unnecessary biopsies when the cutoff was 24. An increasing number of scholars have argued that the combination of PHI and PI-RADS could avoid more unnecessary biopsies than PHI or PI-RADS alone. In a prospective study, researchers found that the AUC of the combination of PHI and PI-RADS was higher than that of PHI (0.873 vs. 0.735, *p* = 0.002) or PI-RADS (0.873 vs. 0.830, *p* = 0.035) alone, and this study showed the superiority of combined diagnosis (22). There were also some studies that compare different cutoff values in the model of combined diagnosis and found the cutoff value that can save the maximum degree of biopsies (23). However, in their series, these studies only compared the single predictor with the multivariable model and did not establish the base model with some primary indicators (such as age, BMI, TPSA, and the medical history of DM and hypertension). In our study, the AUCs of different models, which were developed on the basis of TPSA derivatives, PHI derivatives, PI-RADS, and the combination of PHI and PI-RADS, were compared for the detection of PCa and CSPCa. For the detection of PCa in TPSA 4–20 ng/ml, the multivariable combined model could avoid 73.97% unnecessary biopsies compared with 45.21% unnecessary biopsies in the model based on PHI at a sensitivity of 95%.

In this study, univariable and multivariable regression analyses were carried out between the predictors and GS, and a nonlinear pattern between f/T and PV was found. Therefore, f/T was excluded in the stepwise logistic regression. This also showed contradictory outcomes in detecting PCa (21, 24). To avoid the exclusion of f/T, we use the logarithmic transformation and included the log(f/T) into models. When PV alone was used as a predictor to predict the diagnostic accuracy of PCa and CSPCa, it showed comparatively low prediction accuracy. Log (PSAD) performed better than the single indicator for predicting PCa and CSPCa in our study. However, Log (PSAD) was excluded from the stepwise logistic regression analysis, TPSA and PV were finally included in our model. This may be an independent predictor that could better establish multivariable models (25). The AUC of PHI for detecting CSPCa was higher than the AUC for PCa, which indicated superiority in diagnosing aggressive PCa. This proven PHI was beneficial to the diagnosis of CSPCa compared with other indicators (26, 27). When comparing PHI with PHID, PHI performed with better predictive accuracy than PHID for CSPCa (28).

Adding PI-RADS V2.1, which was assessed by mpMRI, to PHI could improve the diagnosis of PCa and CSPCa. The mpMRI can identify abnormal signals, and urologists can perform targeted biopsies for abnormal signals, which can find more CSPCa than systematic 12-core transrectal ultrasound-guided biopsy (29, 30). Luzzago et al. proposed that patients could avoid prostate biopsies when they had negative mpMRI tests, and only a small percentage of them (4.1%) would be found to have CSPCa during the follow-up with repeated PSA tests. In our study, the AUC of the model developed on the basis of TPSA derivatives and PI-RADS was significantly lower than the AUC of the model with PHI and PI-RADS (*p* = 0.004 and *p* = 0.003 for PCa and CSPCa), which laterally reflected the superiority of the application of PHI. If these researchers applied PHI to their studies (30), more unnecessary biopsies may be avoided. In univariable and multivariable logistic regression analyses, the value of OR was largest compared with the values of other predictors, which showed that mpMRI was a certain risk factor in detecting PCa and CSPCa (31).

In addition, it is worthwhile to consider the role of PHI and PI-RADS in various races. According to a study of Caucasian men, PHI significantly improved PCa detection in magnetic resonance imaging-guided transrectal targeted prostate biopsy and showed the highest AUC (0.79) compared to other PSA derivatives (32). While the study did not directly compare the effects of PHI combined with PI-RADS, it indirectly illustrated the importance of combining PHI and mpMRI, which indicated that the two indicators can also be beneficial to white and Caucasian populations. A study from Loyola and UAB populations showed that Asian American men have a lower risk of PCa (OR = 0.15, 95% CI: 0.06–0.42) and CSPCa (OR = 0.34, 95% CI: 0.12–1.02) than other races (33). For men with African ancestry, Patel et al. conducted a study evaluating implications of race (African American men versus non-African American men) and tumor location for PCa detection (34). The authors concluded that African American men did not find statistically significant difference in the number of lesions, number of anterior prostate lesions, or distribution of highest PI-RADS lesions but had a higher risk of PCa, which indicated that the risk of PCa in African American men cannot be explained simply by PI-RADS score distribution and tumor location (34). Furthermore, the patients of this cohort were biopsy-naïve, and the biopsy setting could influence the performance of models. A prospective study involving 900 patients conducted by Patel et al. illustrated that patients with prior negative biopsies had lower PCa detection (27.9% vs 54.4%) in comparison with biopsy-naive men (35), demonstrating the importance of the biopsy setting in cancer detection.

We also found other statistically significant indicators; age and BMI also performed significantly in detecting CSPCa. This finding indicated that older men could suffer from aggressive PCa (36, 37). The BMI of patients with CSPCa was significantly higher than that of patients with non-CSPCa, which showed that obesity may lead to aggressive PCa (38). Some studies have proposed that DM is inconsistent with PCa. Tao et al. found that DM was a significant risk factor for predicting PCa. However, a review proposed that the morbidity of men with DM was lower than that of men without DM [OR, 0.80; 95% confidence interval (CI), 0.76–0.85] (39). In terms of hypertension, a meta-analysis of metabolic syndrome and PCa proposed that hypertension was associated with the risk of PCa (40). However, in our study, we found no significant difference between PCa and non-PCa in DM and hypertension, which suggests that more evidence should be provided to confirm the relationship between DM, hypertension, and PCa.

Several limitations still exist in our study. First, the study was a retrospective and single-center cohort study. Owing to the retrospective nature and the lack of external validation, the predictive ability of the multivariable models remains to be confirmed using prospective studies and larger cohorts. Second, through the analysis of PI-RADS alone, we found that the number of patients with PI-RADS = 3 was equal in CSPCa and non-CSPCa, but the proportion of PI-RADS ≥ 4 in CSPCa was higher than that in non-CSPCa, which may be due to the limitation of having a small cohort. Finally, our study evaluated the influence of BMI, DM, and hypertension on PCa, but did not include other components of metabolic syndrome, such as lipids and albumin.

## Conclusion

The models combining PHI derivatives and PI-RADS performed better in detecting PCa and CSPCa than the models based on PHI or PI-RADS individually in TPSA 4–20 ng/ml. It was found that adding the PI-RADS to the PHI-based model significantly improved PCa detection in TPSA 4–10 ng/ml. The addition of PHI to the model based on the PI-RADS was more useful in detecting PCa and CSPCa in TPSA 10–20 ng/ml.

## Data Availability Statement

The original contributions presented in the study are included in the article/**Supplementary Material**. Further inquiries can be directed to the corresponding author.

## Ethics Statement

The studies involving human participants were reviewed and approved by Ethical Committee of the Qilu Hospital of Shandong University. Written informed consent for participation was not required for this study in accordance with the institutional requirements and the national legislation.

## Author Contributions

Study design: YFZ and YHZ. Data collection: YHZ, WQ, and MZ. Data analysis: YHZ, GL, and RL. Drafting the manuscript: YHZ, JC, and SQ. Project supervision: YFZ, BS, and SC. All authors contributed to the article and approved the submitted version.

## Funding

This work was supported by the National Natural Science Foundation of China (Grant Nos. 81970661 and 82170790 to BS) and the Natural Science Foundation of Shandong Province (ZR2021MH318 to YFZ).

## Conflict of Interest

The authors declare that the research was conducted in the absence of any commercial or financial relationships that could be construed as a potential conflict of interest.

## Publisher’s Note

All claims expressed in this article are solely those of the authors and do not necessarily represent those of their affiliated organizations, or those of the publisher, the editors and the reviewers. Any product that may be evaluated in this article, or claim that may be made by its manufacturer, is not guaranteed or endorsed by the publisher.

## Supplementary Material

Supplementary Figure 1 The ROC curves and the curves of correction for multiple comparisons of models. **(A)** The models for PCa in TPSA 4-10 ng/ml. **(B)** The models for CSPCa in TPSA 4-10 ng/ml. **(C)** The models for PCa in TPSA 10-20 ng/ml. **(D)** The models for CSPCa in TPSA 10-20 ng/ml. **(E)** The models for PCa in TPSA 4-20 ng/ml. **(F)** The models for CSPCa in TPSA 4-20 ng/ml. R^1(t): Model A: multivariable model that based on the TPSA derivatives, R^2(t): Model B: multivariable model that based on the combination of PHI derivatives and base model, R^3(t): Model C: multivariable model that based on combination of the PI-RADS and base model, R^4(t): Model D: multivariable model that based on the combination of PHI derivatives and PI-RADS, R^(t): The curve of correction for multiple comparisons.
