# Supplementary figures and images for: Construction and Comparison of Different Models in Detecting Prostate Cancer and Clinically Significant Prostate Cancer
Source: Front Oncol. 2022 Jul 12;12:911725. doi: 10.3389/fonc.2022.911725 (PMC9316170; doi:10.3389/fonc.2022.911725)

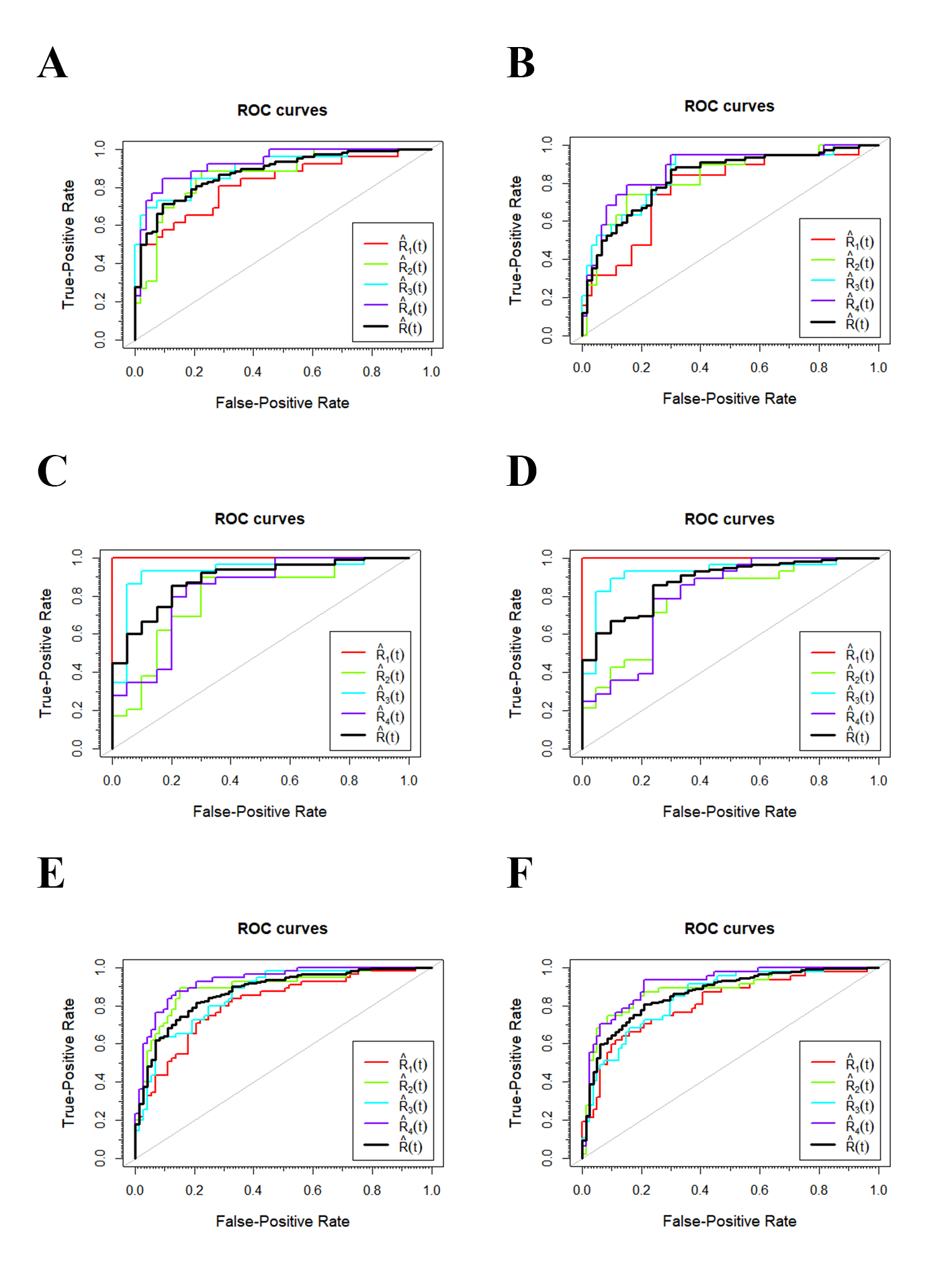

Supplement: Supplementary Figure 1 — The ROC curves and the curves of correction for multiple comparisons of models. (A) The models for PCa in TPSA 4-10 ng/ml. (B) The models for CSPCa in TPSA 4-10 ng/ml. (C) The models for PCa in TPSA 10-20 ng/ml. (D) The models for CSPCa in TPSA 10-20 ng/ml. (E) The models for PCa in TPSA 4-20 ng/ml. (F) The models for CSPCa in TPSA 4-20 ng/ml. R^1(t): Model A: multivariable model that based on the TPSA derivatives, R^2(t): Model B: multivariable model that based on the combination of PHI derivatives and base model, R^3(t): Model C: multivariable model that based on combination of the PI-RADS and base model, R^4(t): Model D: multivariable model that based on the combination of PHI derivatives and PI-RADS, R^(t): The curve of correction for multiple comparisons. [file Image_1.tif]
