# Supplementary material for: Construction and Comparison of Different Models in Detecting Prostate Cancer and Clinically Significant Prostate Cancer
Source: Front Oncol. 2022 Jul 12;12:911725. doi: 10.3389/fonc.2022.911725 (PMC9316170; doi:10.3389/fonc.2022.911725)
Supplement: Supplementary file 2 [file Table_1.docx]

**Table S1a.** Multivariable analysis of the predictive value of the different models in the diagnosis of PCa in the PSA 4-20 ng/ml.

| **Total PCa** | **Model A** | | **Model B** | | **Model C** | | **Model D** | |
| --- | --- | --- | --- | --- | --- | --- | --- | --- |
|  | **OR**  **(95% CI)** | ***P*** | **OR**  **(95% CI)** | ***P*** | **OR**  **(95% CI)** | ***P*** | **OR**  **(95% CI)** | ***P*** |
| Age | 1.103(1.045-1.165) | ＜0.001 | 1.099(1.027-1.175) | 0.006 | 1.069(1.007-1.135) | 0.028 | 1.054(0.979-1.135) | 0.162 |
| BMI | 1.238(1.062-1.443) | 0.006 | 1.298(1.079-1.560) | 0.006 | 1.271(1.074-1.503) | 0.005 | 1.354(1.094-1.677) | 0.005 |
| TPSA | 1.198(1.055-1.361) | 0.005 | 1.017(0.864-1.198) | 0.836 | 1.168(1.021-1.336) | 0.024 | 0.964(0.807-1.151) | 0.684 |
| Log(f/T) | 0.247(0.016-3.797)) | 0.316 | 0.457(0.014-14.484) | 0.657 | 0.287(0.017-4.963) | 0.391 | 1.267(0.029-55.048) | 0.902 |
| PV | 0.974(0.955-0.994) | 0.009 | 0.981(0.959-1.004) | 0.108 | 0.977(0.956-0.998) | 0.032 | 0.984(0.960-1.009) | 0.211 |
| PHI | NA | NA | 1.054(1.028-1.081) | ＜0.001 | NA | NA | 1.061(1.030-1.093) | ＜0.001 |
| PI-RADS | NA | NA | NA | NA | 2.683(1.620-4.442) | ＜0.001 | 3.082(1.597-5.947) | 0.001 |
| AUC (95%CI) | 0.809(0.733-0.885) | | 0.893(0.834-0.952) | | 0.862(0.799-0.924) | | 0.928(0.884-0.972) | |
| *P* (Model A as referent) | / | | 0.003 | | 0.025 | | ＜0.001 | |
| *P* (Model B as referent) | / | | / | | 0.285 | | 0.031 | |
| *P* (Model C as referent) | / | | / | | / | | 0.004 | |

Model A：multivariable model that based on the TPSA derivatives (base model), Model B: multivariable model that based on the combination of PHI derivatives and base model, Model C: multivariable model that based on combination of the PI-RADS and base model, Model D: multivariable model that based on the combination of PHI derivatives and PI-RADS. OR: odds ratio, 95% CI：95% confidence intervals. BMI: body mass index, TPSA: total prostate specific antigen, f/T: free/ Total prostate-specific antigen, PV: prostate volume, PHI: prostate health index, PI-RADS: Prostate Imaging Reporting and Data System version 2.1, PCa: prostate cancer

**Table S1b.** Multivariable analysis of the predictive value of the different models in the diagnosis of CSPCa in the PSA 4-20 ng/ml.

| **Total CSPCa** | **Model A** | | **Model B** | | **Model C** | | **Model D** | |
| --- | --- | --- | --- | --- | --- | --- | --- | --- |
|  | **OR**  **(95% CI)** | ***P*** | **OR**  **(95% CI)** | ***P*** | **OR**  **(95% CI)** | ***P*** | **OR**  **(95% CI)** | ***P*** |
| Age | 1.081(1.025-1.139) | 0.004 | 1.069(1.004-1.138) | 0.037 | 1.050(0.990-1.112) | 0.102 | 1.038(0.970-1.110) | 0.283 |
| BMI | 1.190(1.020-1.390) | 0.027 | 1.217(1.020-1.453) | 0.029 | 1.198(1.015-1.413) | 0.033 | 1.212(1.001-1.466) | 0.048 |
| TPSA | 1.288(1.126-1.473) | ＜0.001 | 1.146(0.979-1.343) | 0.090 | 1.265(1.100-1.456) | 0.001 | 1.127(0.956-1.328) | 0.154 |
| Log(f/T) | 0.239(0.014-4.147) | 0.325 | 0.404(0.012-13.141) | 0.610 | 0.275(0.014-5.244) | 0.391 | 0.603(0.017-21.483) | 0.781 |
| PV | 0.980(0.962-1.000) | 0.045 | 0.988(0.967-1.010) | 0.278 | 0.984(0.964-1.005) | 0.025 | 0.992(0.969-1.014) | 0.472 |
| PHI | NA | NA | 1.041(1.020-1.063) | ＜0.001 | NA | NA | 1.041(1.018-1.065) | ＜0.001 |
| PI-RADS | NA | NA | NA | NA | 2.235(1.375-3.633) | 0.001 | 2.099(1.208-3.647) | 0.009 |
| AUC (95%CI) | 0.812(0.734-0.891) | | 0.884(0.820-0.948) | | 0.846(0.779-0.913) | | 0.913(0.862-0.963) | |
| *P* (Model A as referent) | / | | 0.013 | | 0.137 | | 0.001 | |
| *P* (Model B as referent) | / | | / | | 0.201 | | 0.061 | |
| *P* (Model C as referent) | / | | / | | / | | 0.003 | |

Model A：multivariable model that based on the TPSA derivatives (base model), Model B: multivariable model that based on the combination of PHI derivatives and base model, Model C: multivariable model that based on combination of the PI-RADS and base model, Model D: multivariable model that based on the combination of PHI derivatives and PI-RADS. OR: odds ratio, 95% CI：95% confidence intervals. BMI: body mass index, TPSA: total prostate specific antigen, f/T: free/ Total prostate-specific antigen, PV: prostate volume, PHI: prostate health index, PI-RADS: Prostate Imaging Reporting and Data System version 2.1, CSPCa: clinically significant prostate cancer, defined as Gleason Grade ≥ 2 prostate cancer
