# Supplementary material for: Construction and Comparison of Different Models in Detecting Prostate Cancer and Clinically Significant Prostate Cancer
Source: Front Oncol. 2022 Jul 12;12:911725. doi: 10.3389/fonc.2022.911725 (PMC9316170; doi:10.3389/fonc.2022.911725)
Supplement: Supplementary file 3 [file Table_2.docx]

**Table S2a.** Diagnostic efficiency of different models in PSA 4-10ng/ml.

|  | **Cut-off value** | **Sensitivity (%)** | **Specificity (%)** | **PPV (%)** | **NPV (%)** | **+LR (%)** | **–LR (%)** | **ODA (%)** |
| --- | --- | --- | --- | --- | --- | --- | --- | --- |
| **PCa** |  |  |  |  |  |  |  |  |
| **Model B** |  |  |  |  |  |  |  |  |
|  | 0.094 | 96.15 | 45.28 | 46.30 | 96.00 | 1.76 | 0.09 | 62.03 |
|  | 0.295 | 88.46 | 77.36 | 65.71 | 93.18 | 3.91 | 015 | 81.01 |
|  | 0.323 | 84.62 | 79.25 | 66.67 | 91.30 | 4.08 | 0.19 | 81.01 |
|  | 0.336 | 80.77 | 79.25 | 65.63 | 89.36 | 3.89 | 0.24 | 79.75 |
| **Model C** |  |  |  |  |  |  |  |  |
|  | 0.106 | 96.15 | 56.60 | 52.08 | 96.77 | 2.22 | 0.07 | 69.62 |
|  | 0.180 | 88.46 | 67.92 | 57.50 | 92.31 | 2.76 | 0.17 | 74.68 |
|  | 0.340 | 84.62 | 81.13 | 68.75 | 91.49 | 4.48 | 0.19 | 82.28 |
|  | 0.368 | 80.77 | 81.13 | 67.74 | 89.58 | 4.28 | 0.24 | 81.01 |
| **Model D** |  |  |  |  |  |  |  |  |
|  | 0.085 | 96.15 | 56.60 | 52.08 | 96.77 | 2.22 | 0.07 | 69.62 |
|  | 0.341 | 88.46 | 81.13 | 69.70 | 93.48 | 4.69 | 0.14 | 83.54 |
|  | 0.452 | 84.61 | 90.57 | 81.48 | 92.31 | 8.97 | 0.17 | 88.61 |
|  | 0.465 | 80.77 | 90.57 | 80.77 | 90.57 | 8.57 | 0.21 | 87.34 |
| **CSPCa** |  |  |  |  |  |  |  |  |
| **Model B** |  |  |  |  |  |  |  |  |
|  | 0.109 | 94.74 | 45.00 | 35.29 | 96.43 | 1.72 | 0.12 | 56.96 |
|  | 0.168 | 89.47 | 60.00 | 41.46 | 94.74 | 2.24 | 0.18 | 67.09 |
|  | 0.176 | 84.21 | 60.00 | 40.00 | 92.31 | 2.11 | 0.26 | 65.82 |
|  | 0.247 | 78.95 | 76.67 | 51.72 | 92.00 | 3.38 | 0.27 | 77.22 |
| **Model C** |  |  |  |  |  |  |  |  |
|  | 0.168 | 94.74 | 68.33 | 48.65 | 97.62 | 2.99 | 0.08 | 74.68 |
|  | 0.188 | 89.47 | 70.00 | 48.57 | 95.45 | 2.98 | 0.15 | 74.68 |
|  | 0.213 | 84.21 | 70.00 | 47.06 | 93.33 | 2.81 | 0.23 | 73.42 |
|  | 0.280 | 78.95 | 75.00 | 50.00 | 91.84 | 3.158 | 0.28 | 75.95 |
| **Model D** |  |  |  |  |  |  |  |  |
|  | 0.197 | 94.74 | 70.00 | 50.00 | 97.67 | 3.16 | 0.08 | 75.95 |
|  | 0.220 | 89.47 | 71.67 | 50.00 | 95.56 | 3.16 | 0.15 | 75.94 |
|  | 0.224 | 84.21 | 71.67 | 48.48 | 93.48 | 2.97 | 0.22 | 74.68 |
|  | 0.305 | 78.95 | 85.00 | 62.50 | 92.73 | 5.26 | 0.25 | 83.54 |

PCa: prostate cancer, CSPCa: clinically significant prostate cancer, defined as Gleason Grade ≥ 2 prostate cancer, Model B: multivariable model that based on the combination of PHI derivatives and base model, Model C: multivariable model that based on combination of the PI-RADS and base model, Model D: multivariable model that based on the combination of PHI derivatives and PI-RADS, PPV, positive predictive value; NPV, negative predictive value; +LR, positive likelihood ratio; –LR, negative likelihood ratio; ODA, overall diagnostic accuracy.

**Table S2b.** Diagnostic efficiency of different models in PSA 10-20ng/ml.

|  | **Cut-off value** | **Sensitivity (%)** | **Specificity (%)** | **PPV (%)** | **NPV (%)** | **+LR (%)** | **–LR (%)** | **ODA (%)** |
| --- | --- | --- | --- | --- | --- | --- | --- | --- |
| **PCa** |  |  |  |  |  |  |  |  |
| **Model B** |  |  |  |  |  |  |  |  |
|  | 0.250 | 96.55 | 65.00 | 80.00 | 92.86 | 2.76 | 0.05 | 83.67 |
|  | 0.442 | 89.66 | 90.00 | 92.86 | 85.71 | 8.97 | 0.11 | 89.80 |
|  | 0.589 | 86.21 | 95.00 | 96.15 | 82.61 | 17.24 | 0.15 | 89.80 |
|  | 0.676 | 79.31 | 95.00 | 95.83 | 76.00 | 15.86 | 0.22 | 85.71 |
| **Model C** |  |  |  |  |  |  |  |  |
|  | 0.278 | 96.55 | 45.00 | 71.79 | 90.00 | 1.76 | 0.08 | 75.51 |
|  | 0.446 | 89.66 | 65.00 | 78.79 | 81.25 | 2.56 | 0.16 | 79.59 |
|  | 0.557 | 86.21 | 75.00 | 83.33 | 78.95 | 3.45 | 0.18 | 81.63 |
|  | 0.612 | 79.31 | 80.00 | 85.19 | 72.73 | 3.97 | 0.26 | 79.59 |
| **Model D** |  |  |  |  |  |  |  |  |
|  | 0.329 | 96.55 | 75.00 | 84.85 | 93.75 | 3.86 | 0.05 | 87.76 |
|  | 0.572 | 89.66 | 95.00 | 96.30 | 86.36 | 17.93 | 0.11 | 91.84 |
|  | 0.641 | 86.21 | 95.00 | 96.15 | 82.61 | 17.24 | 0.15 | 89.80 |
|  | 0.729 | 79.31 | 95.00 | 95.83 | 76.00 | 15.86 | 0.22 | 85.71 |
| **CSPCa** |  |  |  |  |  |  |  |  |
| **Model B** |  |  |  |  |  |  |  |  |
|  | 0.228 | 96.43 | 57.14 | 75.00 | 92.31 | 2.25 | 0.06 | 79.59 |
|  | 0.422 | 89.29 | 90.48 | 92.59 | 86.36 | 9.38 | 0.12 | 89.80 |
|  | 0.496 | 85.71 | 90.48 | 92.31 | 82.61 | 9.00 | 0.16 | 87.76 |
|  | 0.685 | 78.57 | 95.24 | 95.65 | 76.92 | 16.51 | 0.23 | 85.71 |
| **Model C** |  |  |  |  |  |  |  |  |
|  | 0.307 | 96.43 | 47.62 | 71.05 | 90.91 | 1.84 | 0.07 | 75.51 |
|  | 0.414 | 89.29 | 61.90 | 75.76 | 81.25 | 2.34 | 0.17 | 77.55 |
|  | 0.496 | 85.71 | 66.67 | 77.42 | 77.78 | 2.57 | 0.21 | 77.55 |
|  | 0.569 | 78.57 | 76.19 | 81.48 | 72.73 | 3.30 | 0.28 | 77.55 |
| **Model D** |  |  |  |  |  |  |  |  |
|  | 0.332 | 96.43 | 76.19 | 84.38 | 94.11 | 4.05 | 0.05 | 87.76 |
|  | 0.498 | 89.29 | 95.24 | 96.15 | 86.96 | 18.76 | 0.11 | 91.84 |
|  | 0.511 | 85.71 | 95.24 | 96.00 | 83.33 | 18.01 | 0.15 | 89.80 |
|  | 0.683 | 78.57 | 95.24 | 95.65 | 76.92 | 16.51 | 0.23 | 85.71 |

PCa: prostate cancer, CSPCa: clinically significant prostate cancer, defined as Gleason Grade ≥ 2 prostate cancer, Model B: multivariable model that based on the combination of PHI derivatives and base model, Model C: multivariable model that based on combination of the PI-RADS and base model, Model D: multivariable model that based on the combination of PHI derivatives and PI-RADS, PPV, positive predictive value; NPV, negative predictive value; +LR, positive likelihood ratio; –LR, negative likelihood ratio; ODA, overall diagnostic accuracy.

**Table S2c.** Diagnostic efficiency of different models in PSA 4-20ng/ml.

|  | **Cut-off value** | **Sensitivity (%)** | **Specificity (%)** | **PPV (%)** | **NPV (%)** | **+LR (%)** | **–LR (%)** | **ODA (%)** |
| --- | --- | --- | --- | --- | --- | --- | --- | --- |
| **PCa** |  |  |  |  |  |  |  |  |
| **Model B** |  |  |  |  |  |  |  |  |
|  | 0.120 | 94.55 | 45.21 | 56.52 | 91.67 | 1.73 | 0.12 | 66.41 |
|  | 0.250 | 90.91 | 67.12 | 67.57 | 90.74 | 2.76 | 0.14 | 77.34 |
|  | 0.396 | 85.45 | 84.93 | 81.03 | 88.57 | 5.67 | 0.17 | 85.16 |
|  | 0.482 | 80.00 | 86.30 | 81.48 | 85.14 | 5.84 | 0.23 | 83.59 |
| **Model C** |  |  |  |  |  |  |  |  |
|  | 0.237 | 94.55 | 58.90 | 63.41 | 93.48 | 2.30 | 0.09 | 74.22 |
|  | 0.315 | 90.91 | 63.01 | 64.94 | 90.20 | 2.46 | 0.14 | 75.00 |
|  | 0.343 | 85.45 | 67.12 | 66.20 | 85.96 | 2.60 | 0.22 | 75.00 |
|  | 0.417 | 80.00 | 75.34 | 70.97 | 83.33 | 3.24 | 0.27 | 77.34 |
| **Model D** |  |  |  |  |  |  |  |  |
|  | 0.288 | 94.55 | 73.97 | 73.24 | 94.74 | 3.63 | 0.07 | 82.81 |
|  | 0.382 | 90.91 | 79.45 | 76.92 | 92.06 | 4.42 | 0.11 | 84.38 |
|  | 0.447 | 85.45 | 87.67 | 83.92 | 88.89 | 6.93 | 0.17 | 86.72 |
|  | 0.501 | 80.00 | 89.04 | 84.62 | 85.53 | 7.30 | 0.22 | 85.16 |
| **CSPCa** |  |  |  |  |  |  |  |  |
| **Model B** |  |  |  |  |  |  |  |  |
|  | 0.101 | 95.74 | 37.03 | 46.88 | 93.75 | 1.52 | 0.12 | 58.59 |
|  | 0.137 | 91.49 | 46.91 | 50.00 | 90.48 | 1.72 | 0.18 | 63.28 |
|  | 0.312 | 85.11 | 80.25 | 71.43 | 90.28 | 4.31 | 0.19 | 82.03 |
|  | 0.327 | 80.85 | 82.72 | 73.08 | 88.16 | 4.68 | 0.23 | 82.03 |
| **Model C** |  |  |  |  |  |  |  |  |
|  | 0.198 | 95.74 | 54.32 | 54.88 | 96.65 | 2.10 | 0.08 | 69.53 |
|  | 0.251 | 91.49 | 64.20 | 59.72 | 92.86 | 2.56 | 0.13 | 74.22 |
|  | 0.282 | 85.11 | 69.14 | 61.54 | 88.89 | 2.76 | 0.22 | 75.00 |
|  | 0.322 | 80.85 | 70.37 | 61.29 | 86.36 | 2.73 | 0.27 | 74.21 |
| **Model D** |  |  |  |  |  |  |  |  |
|  | 0.139 | 95.74 | 58.02 | 56.96 | 95.92 | 2.28 | 0.07 | 71.88 |
|  | 0.264 | 91.49 | 79.01 | 71.67 | 94.11 | 4.36 | 0.11 | 83.59 |
|  | 0.287 | 85.11 | 80.25 | 71.43 | 90.28 | 4.31 | 0.19 | 82.03 |
|  | 0.334 | 80.85 | 83.95 | 74.51 | 88.31 | 5.04 | 0.23 | 82.81 |

PCa: prostate cancer, CSPCa: clinically significant prostate cancer, defined as Gleason Grade ≥ 2 prostate cancer, Model B: multivariable model that based on the combination of PHI derivatives and base model, Model C: multivariable model that based on combination of the PI-RADS and base model, Model D: multivariable model that based on the combination of PHI derivatives and PI-RADS, PPV, positive predictive value; NPV, negative predictive value; +LR, positive likelihood ratio; –LR, negative likelihood ratio; ODA, overall diagnostic accuracy.
